# Diagnosis and Management of Extracranial Head and Neck Schwannomas: A Review of 27 Cases

**DOI:** 10.1155/2013/973045

**Published:** 2013-05-08

**Authors:** Ryuji Yasumatsu, Torahiko Nakashima, Rina Miyazaki, Yuichi Segawa, Shizuo Komune

**Affiliations:** Department of Otorhinolaryngology, Graduate School of Medical Sciences, Kyushu University, 3-1-1 Maidashi, Higashi-ku, Fukuoka 812-8582, Japan

## Abstract

*Objectives*. Clinical records of 27 patients with extracranial head and neck schwannoma were retrospectively reviewed. *Methods*. Ultrasonography (US) was performed in all cases. Seven patients underwent CT. Twenty-five patients underwent MRI. Fine needle aspiration cytology (FNAC) was performed for 12 of the 27 patients. Clinical history, surgical data, and postoperative morbidity were analyzed. *Results*. The images of US showed a well-defined, hypoechoic, primarily homogeneous solid mass. At CT, only one of 7 cases (14%) was able to suggest the diagnosis of schwannoma. At MRI, twenty of 25 cases (80%) suggested the diagnosis of schwannoma. Only three of 12 cases (25%) displayed a specific diagnosis of schwannoma rendered on FNAC. The distribution of 27 nerves of origin was 10 (37%) vagus nerves, 6 (22%) sympathetic trunks, 5 (19%) cervical plexuses, 3 (11%) brachial plexuses, 2 (7%) hypoglossal nerves, and 1 (4%) accessory nerve. Complete tumor resection was performed in 11 patients, and intracapsular enucleation of the tumor was performed in 16 patients. The rate of nerve palsy was 100 (11/11) and 31% (5/16). *Conclusions*. MRI is sensitive and specific in the diagnosis of schwannoma. Intracapsular enucleation was an effective and feasible method for preserving the neurological functions.

## 1. Introduction 

Schwannoma is a benign neural sheath tumor, and it occurs in overall body areas including the head and neck region. As a slowly growing benign tumor, it has been reported that 25 to 45% of schwannomas were located in the extracranial head and neck region [1]. It involves the cranial nerves such as V, VII, X, XI, and XII or sympathetic and peripheral nerves [2].

Preoperative diagnostic investigations included ultrasonography (US), computed tomography (CT), magnetic resonance imaging (MRI), and fine needle aspiration cytology (FNAC) [3–5]. However, the preoperative diagnosis of schwannoma is difficult and should be suggested by clinical features and supported by investigations.

As for the management of schwannomas, multiple treatment options exist including observation, complete tumor excision, and intracapsular enucleation [6, 7]. For tumors arising from the major cranial nerves, complete tumor resection renders lifelong morbidity to the patients. On the other hand, the nerve-preserving excision method, such as intracapsular enucleation, does not guarantee intact nerve function after surgery. Because of the substantial chance of nerve palsy after operation, obtaining an accurate preoperative diagnosis, and preferably, with the identification of the nerve of origin is crucial to the management of the disease.

In the present study, clinical records of 27 cases with extracranial head and neck schwannoma treated at our department were retrospectively reviewed.

## 2. Methods

Between 2003 and 2010, 27 patients with extracranial head and neck schwannoma were operated on in the Department of Otorhinolaryngology at Kyushu University Hospital. The data for the 27 patients, consisting of 14 males and 13 females, were analyzed. The subjects' ages ranged from 21 to 80 years, with a median age of 51 years. All cranial nerves were normal, and no Horner's syndrome was noted. Clinical history, surgical data, and postoperative morbidity were obtained. US were performed in all cases. Seven patients underwent CT with or without MRI. Twenty-five patients underwent MRI. Fine needle aspiration cytology (FNAC) was performed for 12 of the 27 patients after imaging. Tumor location, size, and demographic data are described in Table 1. The medical records of these patients were reviewed. 

## 3. Results 

### 3.1. Imaging Findings

The images of US typically showed a well-defined, ovoid or round, hypoechoic, and primarily homogeneous solid mass with or without a moderate posterior acoustic enhancement. None of them showed a direct connection to the nerve.

Seven of 27 patients underwent CT. Five patients (71%) had tumors that were hypoattenuated, with poor enhancement compared with adjoining skeletal muscles. Two tumors (29%) were isoattenuated to skeletal muscle. Only one of seven cases (14%) was able to suggest the diagnosis of schwannoma. 

**Figure 1:**
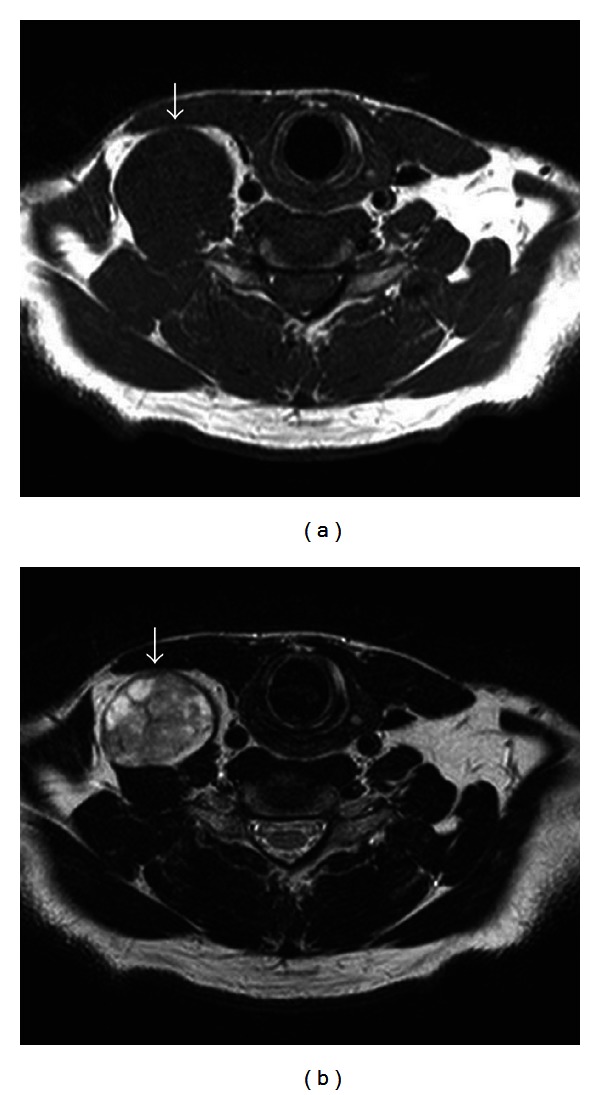
MRI findings for case 24. (a) Axial T1-weighted imaging showed a mass with signal hypointensity (arrow). (b) Axial T2-weighted imaging showed a mass with heterogeneous signal hyperintensity (arrow).

At MRI, all 25 schwannomas revealed relatively low signal intensity on T1-weighted imaging and signal hyperintensity on T2-weighted imaging, with 11 tumors (44%) showing homogeneously high intensity, and 14 tumors (56%) showing heterogeneously high intensity. There were no flow voids seen in any of the tumors. Twenty (80%) suggested the diagnosis of schwannoma.  Figure 1 demonstrates the characteristic features of schwannomas on T1- and T2-weighting MRI. Depending on the site, a number of differential diagnoses were suggested including carotid body tumor, branchial cervical cyst, submandibular tumor, and metastases.

### 3.2. Fine Needle Aspiration Cytology (FNAC)

From these 27 patients, 12 received fine needle aspiration cytology. Only three cases (25%) displayed a specific diagnosis of schwannoma rendered on preoperative FNAC. 

### 3.3. Treatment and Neural Function Outcome

**Figure 2:**
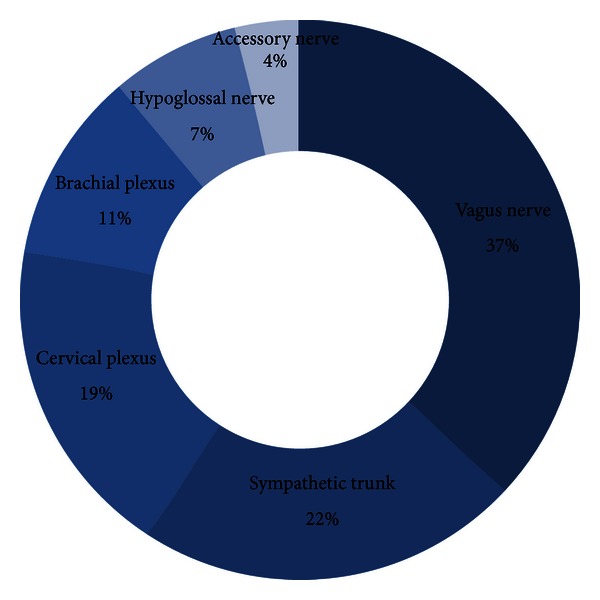
The nerve of origin of 27 extracranial head and neck schwannomas.

All of the tumors were resected through a transcervical approach. The nerve of origin was mainly determined by the postoperative neurological findings. The distribution of 27 nerve of origins was 10 (37%) vagus nerves, 6 (22%) sympathetic trunks, 5 (19%) cervical plexuses, 3 (11%) brachial plexuses, 2 (7%) hypoglossal nerves, and 1 (4%) accessory nerve (Figure 2). 

**Figure 3:**
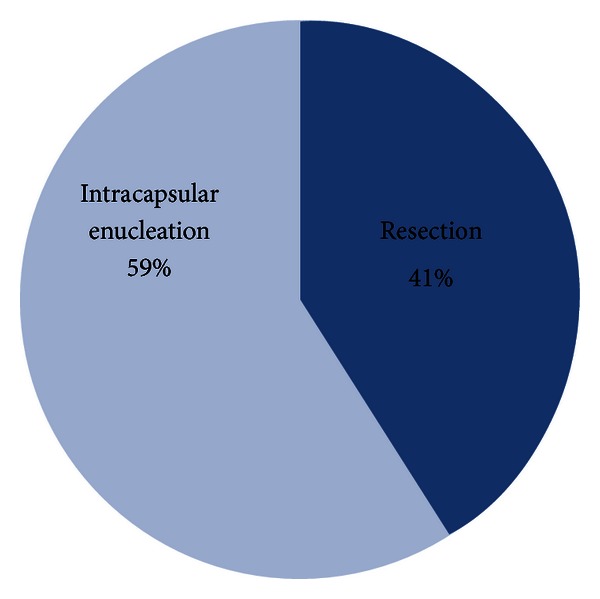
Operation method.

Complete tumor resection was performed on 11 patients, and intracapsular enucleation of the tumor was performed on 16 patients (Figure 3). The preoperative and postoperative neurological functions were evaluated. The rate of nerve palsy at 6 months after complete tumor resection and intracapsular enucleation was 100 (11/11) and 31% (5/16), respectively. In the cases treated with intracapsular enucleation, only one case (20%) maintained normal postoperative neurological function of the five vagal schwannomas. Of the two sympathetic schwannomas, one case (50%) maintained normal postoperative neurological function. In the case of cervical plexus, brachial plexus, and accessory nerve schwannomas, there were no aggravated neurological deficits. In the cases with postoperative nerve palsy treated by intracapsular enucleation, 6 of 11 cases recovered from the palsy within 6 months after operation (Table 2).

## 4. Discussion

Schwannomas are benign tumors that originate from the Schwann cells of the nerve sheath. Schwann cells are neural crest-derived glial cells that are responsible for providing myelin insulation to peripheral nervous system axons [8]. There are several important issues relating to the diagnosis and management of these tumors.

The first of these is difficulty with obtaining a preoperative diagnosis, since symptoms are usually nonspecific [9]. Symptoms, such as severe pain or cranial nerve palsy, would be unusual for these tumors. On examination, these benign masses are typically palpable. In treating schwannoma patients, it is critical to determine the origin of the tumor to preserve nerve function. Some authors suggest that preoperative evaluation with imaging modalities like CT and MRI in determining the nerve of origin may reduce the postoperative neural deficits [5, 10]. 

In terms of preoperative investigations, FNAC, US, and radiographic imaging with CT or MRI are usually performed. However, schwannomas are frequently difficult to characterize on FNAC. Liu et al. reported that the accuracy of FNAC was only 20% [11]. Our results also showed that only three cases (25%) displayed a specific diagnosis of schwannoma. It was not found to be of help in diagnosis.

In the current study, US, was performed in all cases. King et al. showed that schwannomas are highly vascular tumors with an abundance of vessels and blood flow, and the direct connection to the nerve is specific to neurogenic tumors [12]. Although two of five cases showed a direct connection to the nerve in other literature [5], these findings were not detected on US in our cases and were not sensitive enough to use this method. 

**Figure 4:**
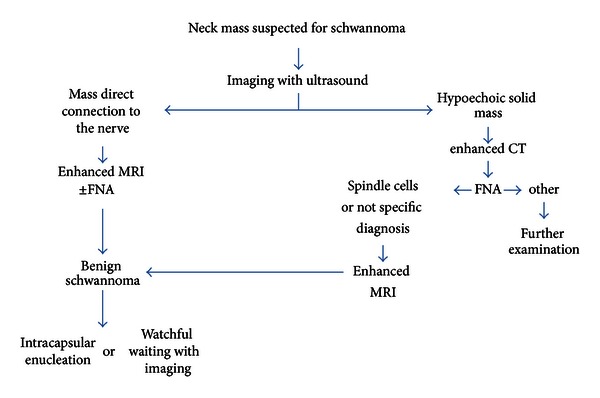
Diagnostic and treatment algorithm for the extracranial head and neck schwannoma.

On noncontrast CT, it was reported that schwannomas were typically hypodense versus muscle; with contrast, these lesions tended to show some peripheral enhancement [10]. Only one case (14%) in our study was able to suggest the diagnosis of schwannoma by CT and clinical features. On the other hand, MRI consistently identifies these lesions on both T1- and T2-weighted imaging. T1-weighted images display low signal intensity, and T2-weighted images show high intensity [5, 10, 13]. Hirano et al., also reported that MRI was especially useful for the diagnosis and peripheral hyperintense rim with central low intensity on enhanced T1 images of MRI [14]. The relationship between the schwannoma and its nerve of origin can be better appreciated with MRI than CT. In addition, MRI appears to be the investigation of choice for diagnosis and identification of nerve of origin. In our cases, twenty cases (80%) suggested the diagnosis of schwannoma. These results indicate that MRI is most sensitive and specific in the diagnosis of schwannoma [5]. The authors propose an algorithm for the management of extracranial head and neck schwannoma (Figure 4).

The decision of operation should be based on the balance between the risk and benefit of the surgery, that is, the severity of preoperative symptomatology and the anticipated postoperative neurological deficit. Surgical excision is the treatment of choice, but slow growth and the noninvasive nature of schwannomas of the neck also allow an observational approach. The preferred method of removing a schwannoma is intracapsular enucleation. Complications are usually transient and in most cases do not require treatment. According to the study by Valentino et al., intracapsular enucleation while preserving the nerve fibers preserved its function by more than 30% when compared to complete tumor resection [7]. In our cases, the rate of nerve palsy at 6 months after complete tumor resection and intracapsular enucleation was 100% and 31%, and none of them recurred more than two years from the operation. These results suggested that intracapsular enucleation was an effective and feasible method for preserving the neurological functions.

In conclusion, cervical schwannomas are rare neck tumors that are not widely discussed in the core surgical literature. Physicians who evaluate neck masses need to be aware of the diagnostic work-up, surgical treatment, and likely complications of this pathology. In addition, treatments assuring the preservation of neurological functions are needed, since surgical resection may cause fatal nerve damage unlike other tumors. An accurate preoperative diagnosis with identification of the nerve of origin, therefore, allows patients to make an informed decision on whether to undergo operation or observation. In addition, before the surgical procedure, we could explain the possible nerve damages to patients. 

## Figures and Tables

**Table 1:** Demographic data, radiological findings, and fine needle aspiration cytology.

Case	Gender	Age	Nerve origin	Tumor size	CT	MRI	FNAC
1	M	54	Vagus nerve	50 × 42 × 40 mm	ND	Schwannoma	Schwannoma
2	M	40	Vagus nerve	100 × 45 × 40 mm	ND	Glomus tumor or schwannoma	ND
3	M	58	Vagus nerve	45 × 35 × 33 mm	ND	Glomus tumor or schwannoma	ND
4	F	37	Vagus nerve	50 × 40 × 42 mm	ND	Schwannoma	Nondiagnostic
5	F	68	Vagus nerve	80 × 35 × 35 mm	Schwannoma	Schwannoma	ND
6	F	32	Vagus nerve	20 × 18 × 15 mm	Cervical tumor	Schwannoma	Nondiagnostic
7	F	80	Vagus nerve	30 × 25 × 25 mm	Cervical tumor	Schwannoma	ND
8	F	61	Vagus nerve	30 × 28 × 20 mm	Cervical tumor	Schwannoma	Nondiagnostic
9	M	54	Vagus nerve	27 × 25 × 25 mm	Cervical tumor	ND	ND
10	F	49	Vagus nerve	30 × 25 × 25 mm	ND	Glomus tumor or schwannoma	Nondiagnostic
11	M	52	Sympathetic trunk	70 × 35 × 35 mm	ND	Schwannoma	ND
12	M	47	Sympathetic trunk	30 × 28 × 22 mm	ND	Schwannoma	ND
13	M	79	Sympathetic trunk	45 × 25 × 20 mm	ND	Schwannoma	ND
14	F	35	Sympathetic trunk	40 × 30 × 25 mm	ND	Glomus tumor or schwannoma	ND
15	F	54	Sympathetic trunk	30 × 28 × 25 mm	ND	Schwannoma	ND
16	M	62	Sympathetic trunk	35 × 25 × 20 mm	ND	Glomus tumor	ND
17	F	42	Cervical plexus	60 × 35 × 33 mm	ND	Schwannoma	Schwannoma
18	M	50	Cervical plexus	35 × 30 × 30 mm	Cervical tumor	Schwannoma	ND
19	M	21	Cervical plexus	40 × 35 × 33 mm	ND	Schwannoma	Nondiagnostic
20	F	55	Cervical plexus	68 × 45 × 40 mm	ND	Schwannoma	Schwannoma
21	F	54	Cervical plexus	20 × 18 × 15 mm	ND	Schwannoma	Nondiagnostic
22	F	31	Brachial plexus	20 × 15 × 15 mm	ND	Schwannoma	Nondiagnostic
23	M	34	Brachial plexus	30 × 30 × 25 mm	ND	Schwannoma	ND
24	M	60	Brachial plexus	45 × 40 × 25 mm	ND	Schwannoma	Schwannoma
25	M	32	Hypoglossal nerve	50 × 35 × 35 mm	ND	Schwannoma	ND
26	F	57	Hypoglossal nerve	30 × 30 × 25 mm	Submandibullar gland tumor	ND	Nondiagnostic
27	F	69	Accessory nerve	40 × 30 × 30 mm	ND	Schwannoma	ND

ND: not done.

**Table 2:** Neural function outcome after tumor intracapsular enucleation.

Case	Nerve origin	Preoperative status	Postoperrative status	6 months after operation
6	Vagus nerve	Normal	Vocal cord paralysis	Vocal cord paralysis
7	Vagus nerve	Normal	Vocal cord paralysis	Vocal cord paralysis
8	Vagus nerve	Normal	Vocal cord paralysis	Vocal cord paralysis
9	Vagus nerve	Normal	Vocal cord paralysis	Vocal cord paralysis
10	Vagus nerve	Normal	Normal	Normal
14	Sympathetic trunk	Normal	Ptosis	Ptosis
15	Sympathetic trunk	Normal	Ptosis	Normal (improved)
17	Cervical plexus	Normal	Paralysis	Normal (improved)
18	Cervical plexus	Normal	Paralysis	Normal (improved)
19	Cervical plexus	Normal	Normal	Normal
20	Cervical plexus	Normal	Normal	Normal
21	Cervical plexus	Normal	Normal	Normal
22	Brachial plexus	Normal	Paralysis	Normal (improved)
23	Brachial plexus	Normal	Paralysis	Normal (improved)
24	Brachial plexus	Normal	Paralysis	Normal (improved)
27	Accessory nerve	Normal	Normal	Normal
